# *Plasmodium falciparum* Activates CD16^+^ Dendritic Cells to Produce Tumor Necrosis Factor and Interleukin-10 in Subpatent Malaria

**DOI:** 10.1093/infdis/jiy555

**Published:** 2018-10-15

**Authors:** Jessica R Loughland, Tonia Woodberry, Michelle J Boyle, Peta E Tipping, Kim A Piera, Fiona H Amante, Enny Kenangalem, Ric N Price, Christian R Engwerda, Nicholas M Anstey, James S McCarthy, Gabriela Minigo

**Affiliations:** 1 Menzies School of Health Research and Charles Darwin University, Darwin, Australia; 2 Burnet Institute, Melbourne, Victoria, Australia; 3 QIMR Berghofer Medical Research Institute, Brisbane, Australia; 4 Timika Malaria Research Program, Papuan Health and Community Development Foundation, Indonesia; 5 District Health Authority, Timika, Papua, Indonesia; 6 Nuffield Department of Clinical Medicine, University of Oxford, Oxford, United Kingdom

**Keywords:** CD16, dendritic cells, malaria, *Plasmodium falciparum*, TNF

## Abstract

**Background:**

The malaria causing parasite *Plasmodium* subverts host immune responses by several strategies including the modulation of dendritic cells (DCs).

**Methods:**

In this study, we show that *Plasmodium falciparum* skewed CD16^+^ DC cytokine responses towards interleukin (IL)-10 production in vitro, distinct to the cytokine profile induced by Toll-like receptor ligation. To determine CD16^+^ DC responsiveness in vivo, we assessed their function after induced *P falciparum* infection in malaria-naive volunteers.

**Results:**

CD16^+^ DCs underwent distinctive activation, with increased expression of maturation markers human leukocyte antigen (HLA)-DR and CD86, enhanced tumor necrosis factor (TNF) production, and coproduction of TNF/IL-10. In vitro restimulation with *P falciparum* further increased IL-10 production. In contrast, during naturally acquired malaria episode, CD16^+^ DCs showed diminished maturation, suggesting increased parasite burden and previous exposure influence DC subset function.

**Conclusions:**

These findings identify CD16^+^ DCs as the only DC subset activated during primary blood-stage human *Plasmodium* infection. As dual cytokine producers, CD16^+^ DCs contribute to inflammatory as well as regulatory innate immune processes.

## Teaser

This study identified CD16^+^ DCs as the only blood DC subset distinctively activated during primary blood-stage human *Plasmodium* infection. As TNF/IL-10 coproducers, CD16^+^ DCs contribute to early inflammatory processes, yet *P falciparum* restimulation skewed cytokine responses further towards IL-10 production.

Malaria is a major global health problem, causing almost half a million deaths in 2016, the majority of which were attributable to *Plasmodium falciparum* [1]. *Plasmodium falciparum* is known to subvert host immune responses by a range of strategies [2] including the modulation of dendritic cells (DCs) [3, 4]. As professional antigen-presenting cells and sentinels of the immune system, functional DCs are pivotal to the generation of robust immune responses [5]. Human peripheral blood myeloid DCs are composed of 2 classic subsets, CD141^+^ DCs and CD1c^+^ DCs, and a third subset CD16^+^ DCs, characterized by surface expression of CD16/FcγRIII [6, 7]. Despite CD16^+^ DCs comprising half the myeloid DC compartment [8], reports describing their function are limited, and their role in *P falciparum* infection is unknown. How CD16^+^ DCs respond to parasite antigen in vitro, after experimentally induced *P falciparum* infection or during naturally acquired malaria, remains to be defined.

Seminal studies have shown the *Plasmodium* parasite can disrupt monocyte-derived DC function in vitro [9, 10]. More recently, in vitro activation of classic blood DC subsets by *P falciparum*-infected red blood cells (RBCs) has been reported [11], whereas CD16^+^ DCs were not evaluated. In clinical malaria, myeloid DC function in general is compromised [4, 12, 13]. Children with uncomplicated malaria show reduced human leukocyte antigen (HLA)-DR expression on CD16^+^ DCs, yet expression of costimulatory molecule CD86 is increased compared with age-matched uninfected controls [14]. The impact of *P falciparum* infection on CD16^+^ DCs in adults remains unclear.

Historically, the classification of CD16^+^ DCs has been ambiguous, and some degree of controversy remains around whether these cells classify as DC or nonclassic monocytes (recently reviewed in [15]). Initially identified as a blood DC subset by phenotype and function [7], subsequent ontology studies classified these cells as a monocyte subset [16, 17]. However, with the rapid rise of cutting-edge technologies, including spade clustering [18] and single-cell ribonucleic acid sequencing [6], CD16^+^ HLA-DR^low^ cells have been shown to cluster separately from monocytes and verified as a human blood DC subset. As innate immune sentinels, CD16^+^ DCs express a broad range of Toll-like receptors (TLR) including TLR1, TLR2, TLR4, and TLR7 [19], equipping them as ideal immune cells for detecting invading pathogens. Previous studies have reported CD16^+^ DC cytokine production in response to lipopolysaccharide (LPS)/interferon (IFN)-γ stimulation, with CD16^+^ DCs considered an inflammatory DC subset in autoimmune diseases [20] and in human immunodeficiency virus-positive individuals [21]. The CD16^+^ DC cytokine profile in response to parasite stimulation in vitro or ex vivo after *Plasmodium* infection is yet to be reported.

Induced malaria studies, in which malaria-naive volunteers are experimentally infected with *Plasmodium* parasites under close clinical monitoring [22], allow the investigation of human immune responses during early subpatent *Plasmodium* infection. During induced malaria after *P falciparum* sporozoite infection, CD16^+^ DCs increase HLA-DR and CD86 expression [23]. In contrast, during induced blood-stage malaria, we have previously shown that classic DCs have impaired activation [3] and plasmacytoid DCs are nonresponsive [24], suggesting that CD16^+^ DCs may respond differently to very early blood-stage *P falciparum* infection. In this study, we describe for the first time the effect of *Plasmodium* parasites on human blood CD16^+^ DC function. We examine CD16^+^ DC maturation and cytokine responses during induced blood-stage *P falciparum* malaria (IBSM) and after in vitro stimulation. We compare these responses to CD16^+^ DC activation in Indonesian children and adults with acute *P falciparum* malaria.

## METHODS

### Induced Blood-Stage *Plasmodium falciparum* Infection

Thirty-nine volunteers, aged 19–41 years (median, 24 years; interquartile range [IQR], 22–28; 44% female and 56% male), consented to participate in a phase Ib clinical trial testing the efficacy of antimalarial drugs: ACTRN12611001203943, registered November 23, 2011; ACTRN12612000323820, registered March 21, 2012; ACTRN12612000814875, registered August 3, 2012; ACTRN12613000565741, registered May 17, 2013; ACTRN12613001040752, registered September 18, 2013; and NCT02281344, registered October 3, 2014. Blood-stage parasitemia was initiated by inoculation of 1800 *P. falciparum*-infected RBCs (pRBC) as previously described [3]. Blood samples were taken (1) at the same time each day before and (2) during blood-stage infection. Antimalarial drugs were administered when volunteers reached a predetermined parasitemia of ≥1000 parasites/mL (day 7 or 8) (Figure 4A). Flow cytometric assays used fresh whole blood and were processed within 2 hours of collection. The study was approved by the Human Research Ethics Committees of QIMR Berghofer Medical Research, NT Department of Health, and Menzies School of Health Research. Details of the clinical trial are reported elsewhere [3, 25].

### Acute Malaria

Phenotype and activation of CD16^+^ DCs were assessed in cryopreserved peripheral blood mononuclear cells (PBMCs) collected from adults and children with uncomplicated *P falciparum* malaria as part of artemisinin combination therapy efficacy studies in Southern Papua, Indonesia [26]. The PBMC samples were collected before commencing treatment and 28 days after antimalarial drug treatment and parasite clearance (Table 1). Written informed consent was obtained from participants. The study was approved by the Human Research Ethics Committees of the National Institute of Health Research and Development, Indonesian Ministry of Health (Jakarta, Indonesia), the NT Department of Health, and Menzies School of Health Research.

**Table 1.** Acute Malaria Patient Characteristics

	UM Adults	Convalescent Adults	UM Children	Convalescent Children
Number of patients	9	11	6	6
Age	30 [23–37]	30 [20–36]	10 [7–11]	10 [9–11]
Male, number (%)	6 (66)	8 (72)	2 (33)	2 (33)
Parasitemia (parasites/µL)	2275 [1167–6048]	NA	7377 [3128–14516]	NA
HRP2 (ng/mL)^a^	9.8 [0.3–41]	NA	53 [1.5–539]	NA
WCC (10^9^/L)	5.05 [4.05–6.23]	NA	5.9 [5.4–7.8]	NA

Values show the median and [interquartile range] unless indicated otherwise.

^a^All samples below detection limit were assigned the value 0.3, which represents half the detection limit.

### Whole Blood and Peripheral Blood Mononuclear Cells CD16^+^ Dendritic Cells Enumeration

CD16^+^ DCs were characterized as lineage^-^ (CD3, CD14, CD19, CD20, CD56, CD34), HLA-DR^+^, CD11c^+^, CD123^−^, and CD16^+^. In brief, 200 µL whole blood or 3M PBMCs were stained with the following surface antibodies: CD3 (HIT3a), CD14 (HCD14), CD19 (HIB19), CD20 (2H7), CD34 (561), CD56 (HCD56), HLA-DR (L243), CD11c (B-Ly6), CD123 (6H6), CD16 (3G8), and CD86 (2331). All antibodies were purchased from BD Biosciences or BioLegend. For whole blood, RBCs were lysed with FACS lysing solution (BD Biosciences) and cells were fixed with 1% (w/v) paraformaldehyde in phosphate-buffered saline (PBS). Absolute numbers of CD16^+^ DCs were determined by adding automated lymphocyte and monocyte counts (10^9^ cells/L), dividing the sum by 100, multiplying the percentage of CD16^+^ DCs, and multiplying the product by 1000 to give cell count/µL. All assays were performed within 2 hours after blood collection because CD16 expression was partially lost from CD16^+^ DCs when blood was kept overnight at ambient temperature.

### Intracellular Cytokine Staining

CD16^+^ DC cytokine production was assessed in 1 mL fresh whole blood unstimulated or stimulated with TLR agonists: TLR1 - Pam3CSK4 100 ng/mL; TLR2 - HKLM 10^8^ cells/mL; TLR4 - *Escherichia coli* K12 LPS 200 ng/mL; or TLR7 - imiquimod 2.5 µg/mL (Sigma-Aldrich), pRBC, or uninfected RBC (uRBC) prepared as previously described [3]; final concentration of 5 M/mL. Protein transport inhibitor (Brefeldin A, GolgiPlug [BD Biosciences]) was added after 2 hours at 37°C, 5% CO_2_. At 6 hours, cells were stained to identify CD16^+^ DCs: CD14^−^ (M5E2), lineage (CD3 [HIT3a], CD19 [HIB19], CD56 [HCD56])^−^, CD1c^−^ (L161), HLA-DR^+^ (L243), CD86^+^ (IT2.2). IgG2b isotype control was used to aid identification of CD16^+^ DCs. The RBCs were lysed with FACS lysing solution (BD Biosciences), washed with 2% fetal calf serum/PBS, and cells permeabilized with ×1 Perm/Wash (BD Biosciences) and stained with intracellular anti-tumor necrosis factor (TNF)-α (MAB11), interleukin (IL)-12/IL-23p40 (C11.5), IL-10 (JES3-9D7), or IgG1 isotype controls (BioLegend). To determine the stimulant-specific response, spontaneous cytokine production was subtracted from responses to pRBC, uRBC, or TLR agonists.

To verify CD16/FcγRIII loss during culture, we assessed DC subset phenotype in 6 unexposed controls. Fresh whole blood staining and the intracellular cytokine assay were performed using the same methods detailed above. Verification of gating strategy used the same antibodies detailed above. FACS data were acquired using a FACSCanto II (BD Biosciences) or Gallios (Beckman Coulter), and data were analyzed using Kaluza 1.3 (Beckman Coulter).

### Statistics

Statistical analyses used GraphPad Prism 6 (Graphpad Software Inc.) and SPICE version 5.3 ([M. Roederer, Vaccine Research Center, National Institute of Allergy and Infectious Diseases, National Institutes of Health] available at: http://exon.niaid.nih.gov) [27]. The Wilcoxon matched-pairs signed-rank test was used to compare longitudinal data. Tests were 2-tailed and considered significant if *P* < .05.

## RESULTS

### Alternative Gating Strategy to Identify CD16^+^ Dendritic Cells After Short-Term Culture

**Figure 1.**
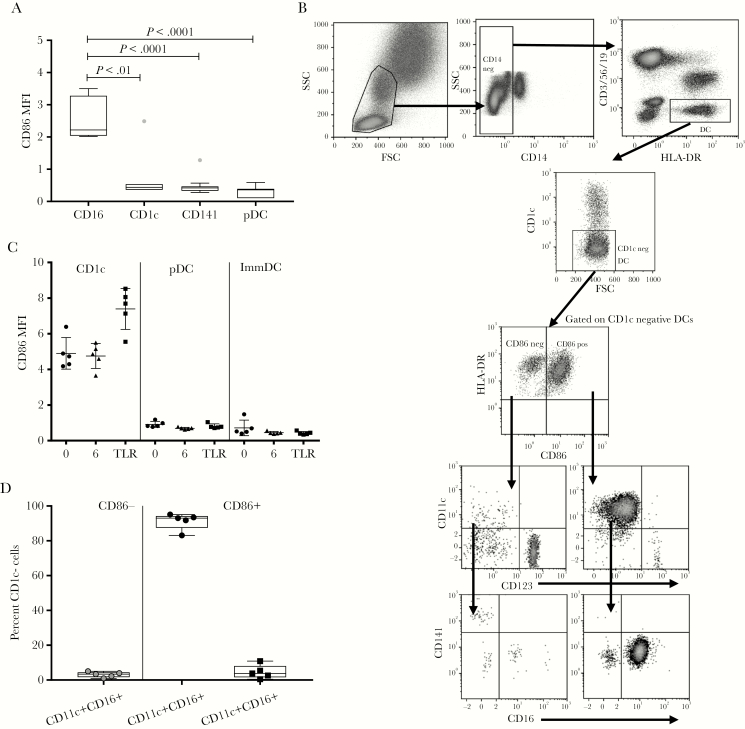
CD16^+^ dendritic cells (DCs) lose CD16 (FcγRIII) expression after short-term culture and Toll-like receptor (TLR)4 stimulation. (A) CD86 expression on circulating blood DC subsets in healthy donors; n = 7 (CD16^+^ DCs and CD1c^+^ DCs), n = 14 (CD141^+^ DCs and pDCs). (B) Representative fresh whole blood gating strategy from a healthy donor to confirm the majority of CD14^−^CD1c^−^HLA-DR^dim^CD86^+^ DC are CD16^+^ DC. First, CD14^+^ cells were excluded, then CD16^+^ DCs were identified as lineage (CD3, CD56, and CD19)^−^, HLA-DR^+^, and CD1c^−^. CD1c^−^ DCs were identified as CD86^−^ DCs or CD86^+^ DCs. The CD86^−^ gate comprised plasmacytoid DCs (pDCs), CD141^+^ DCs, and immature CD11c^−^ DCs, with no CD16^+^ DCs identified. (C) CD86 median fluorescence intensity (MFI) on CD1c^+^ DCs, pDCs, and immature DCs in fresh whole blood (circles), after 6-hour culture (triangles) and after 6-hour culture with TLR4 stimulation (squares), n = 6. Box plots show the 10th-90th percentile, median, and interquartile range for data from all participants. (D) The CD86^+^ gate predominantly (median, 93%; IQR, 88%–94%) comprised CD11c^+^CD16^+^ DCs, and there were no CD16^+^ DCs in the CD1c^−^ CD86^−^ DCs gate, n = 6. FSC, forward scatter; SSC, side scatter.

Measuring cytokine production by CD16^+^ DCs is hampered by the observation that CD16 is lost from the cell surface during short-term culture (6 hours) [28] and after TLR stimulation [29]. To circumvent this problem and identify CD16^+^ DCs, we determined a gating strategy using CD86, a costimulatory molecule highly expressed on CD16^+^ DCs when compared with other blood DC subsets (Figure 1A). By excluding CD1c^+^ DCs from the total DC population and utilizing the differential expression of CD86 on the remaining DC subsets (Figure 1B), we could identify CD16^+^ DCs successfully in fresh whole blood, after short-term culture, thus bypassing the need for CD16 as a critical marker. CD86 expression was negligible on plasmacytoid DCs and immature DCs and did not change after 6 hours of in vitro culture or after TLR stimulation (Figure 1C). CD1c^+^ DCs increased CD86 expression after TLR stimulation, as previously reported [3] (Figure 1C), and were specifically excluded from our alternative CD16^+^ DC gate (Figure 1B). Monocytes were also strictly gated out based on CD14 expression, after confirming that under our culture conditions CD14 median fluorescence intensity on monocytes did not significantly drop within 6 hours after TLR4 stimulation (data not shown). The devised alternative gating strategy selected CD3^−^/CD19^−^/CD56^−^/CD14^−^/HLADR^+^/CD1c^−^/CD86 bright cells, which were predominantly CD16 positive (median, 93%; IQR, 88%–94%) (Figure 1D). Only very few CD16^+^ DCs fell into the CD86-negative gate (Figure 1D). It is noteworthy that if whole blood was kept at room temperature overnight, CD16/FcγRIII was partially lost on CD16^+^ DCs (data not shown), highlighting the importance of performing DC assays on fresh whole blood as soon as possible.

### Cytokine Production by CD16^+^ Dendritic Cells in Response to Toll-Like Receptor and *Plasmodium* Stimulation

**Figure 2.**
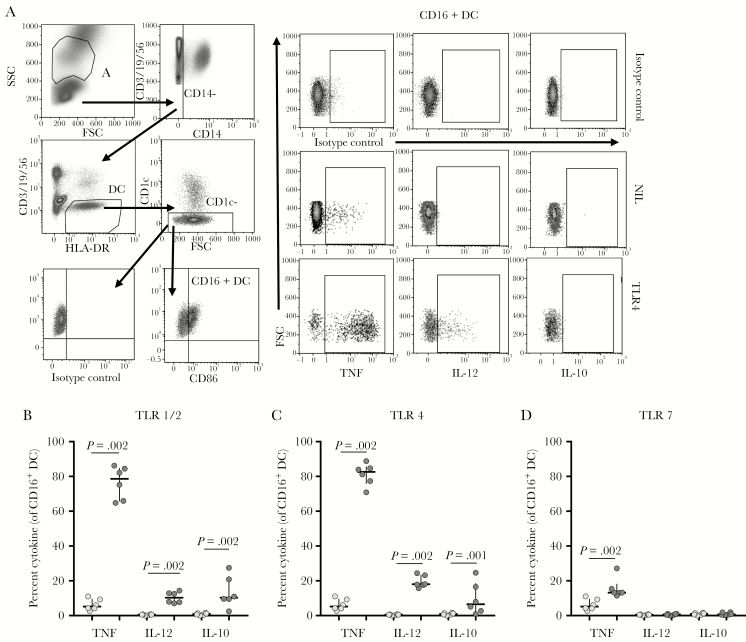
Cytokine profile of CD16^+^ dendritic cells (DCs) in response to in vitro Toll-like receptor (TLR) stimulation. (A) Representative gating of whole blood nonclassical DCs from a naive donor analyzed for intracellular cytokine production (tumor necrosis factor [TNF], interleukin [IL]-12, and IL-10) at 6 hours of culture after stimulation with TLR4 or without stimulation (NIL). CD16^+^ DCs were identified as CD14^−^, lineage (CD3, CD19, and CD56)^−^, human leukocyte antigen-DR^+^, and CD1c. CD1c^−^ nonclassic DCs were gated according to the CD86 isotype control (IgG2b). Cytokine-secreting cells were gated using the appropriate isotype control. (B) CD16^+^ DC cytokine production in response to TLR1/2 (C) TLR4 or (D) TLR7 stimulation. Toll-like receptor stimulations (dark gray circles) were compared with nonstimulation controls (light gray circles). Mann-Whitney *t* test was used for comparison between “no-stim” and TLR. Tests were 2-tailed and considered significant if *P* < .05. FSC, forward scatter; SSC, side scatter.

In fresh whole blood, we assessed CD16^+^ DC cytokine production in response to TLR ligands, using the above gating strategy (Figure 2A). In response to TLR1/2 (PamCys2) or TLR4 (LPS) stimulation, CD16^+^ DCs produced a strong TNF response (74% [IQR, 59%–78%] and 77% [IQR, 71%–79%], respectively), a modest IL-12 response (10% [IQR, 7%–13%] and 17% [IQR, 17%–23%], respectively), and a modest IL-10 response (10% [IQR, 7%–21%] and 6% [IQR, 2%–17%], respectively) (Figure 2B and C). In contrast, after TLR7 (imiquimod) stimulation, most CD16^+^ DCs were nonresponsive, with only 8% (IQR, 2%–15) producing TNF. There was no detectable TLR7-triggered IL-12 or IL-10 response (Figure 2D). Boolean gating was used to assess coproduction of multiple cytokines. Upon TLR1/2 or TLR4 stimulation, a small proportion of CD16^+^ DCs coproduced TNF/IL-12/IL-10, TNF/IL-12, and TNF/IL-10. However, the majority of responders were TNF single producers (Supplementary Figure 1).

**Figure 3.**
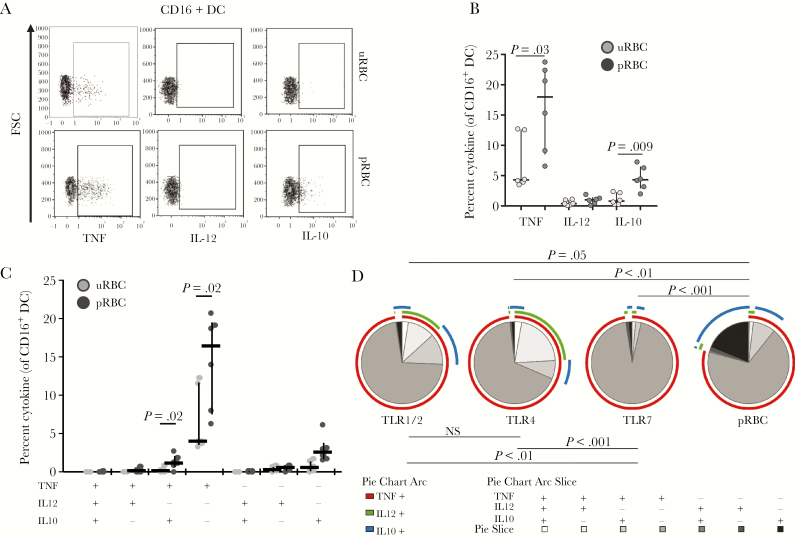
Cytokine profile of CD16^+^ dendritic cells (DCs) in response to in vitro *Plasmodium falciparum*-infected red blood cells (pRBC) stimulation. (A) Representative gating of whole blood nonclassical DCs from a naive donor analyzed for intracellular cytokine production (tumor necrosis factor [TNF], interleukin [IL]-12, and IL-10) at 6 hours of culture after stimulation with unparasitized RBC (uRBC) or pRBC. Gating of nonclassic DCs was performed as per Figure 2A. Mann-Whitney *t* test was used for comparison between uRBC and pRBC. Tests were 2-tailed and considered significant if *P* < .05. (B) CD16^+^ DC cytokine production in response to pRBC stimulation. (C) Boolean gating of the absolute proportion of each combination of TNF, IL-12, or IL-10-producing cells after pRBC stimulation. The pRBC stimulations (dark gray circles) were compared with uRBC controls (light gray circles). (D) The relative proportion (or “composition”) of CD16^+^ DC cytokine responders to Toll-like receptor (TLR) and pRBC stimulation. Each slice of pie represents the median of “a Boolean gate”/“total cytokine producing CD16^+^ DC”. Partial permutation tests were used in SPICE version 5.3.

Next, we assessed responsiveness to pRBC. Upon pRBC stimulation, CD16^+^ DCs significantly increased TNF (*P* = .02) and IL-10 (*P* = .008) production, compared with control cells stimulated with uRBC (Figure 3A and B). Boolean analysis revealed that IL-10 production occurred both as TNF/IL-10-coproducing and IL-10 single-producing cells (Figure 3C). The qualitative composition of cytokine responses (ie, the relative proportion of each cytokine combination amongst the total cytokine producing CD16^+^ DC) after pRBC stimulation significantly differed from evaluated TLR stimulations (Figure 3D). Interleukin-10, either alone or in combination with TNF, comprised a significantly larger relative proportion of the cytokine response after pRBC stimulation compared with TLR stimulation (median fold-increase pRBC over the following: TLR1, 2.9-fold [IQR, 0.7–13.4], *P* = .006; TLR4, 7.8-fold [IQR, 4.1–20.6], *P* = .004; TLR7, 8-fold [IQR, 4.9–16.6], *P* = .004). Together, in vitro stimulation data indicate that CD16^+^ DC cytokine responsiveness is multipotent and stimulation specific.

### Increased Cytokine Production by CD16^+^ Dendritic Cells in Volunteers During Subpatent Induced Blood-Stage Malaria Clinical Trials

**Figure 4.**
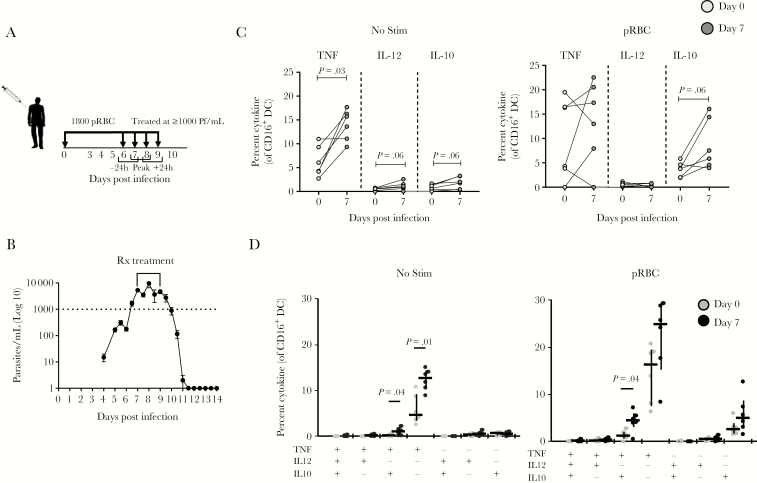
*Plasmodium falciparum* infection and stimulation increases interleukin (IL)-10/tumor necrosis factor (TNF) coproduction by CD16^+^ dendritic cells (DCs). (A) Schematic of the induced blood-stage *P falciparum* malaria (IBSM) clinical trial cohorts, on the days specified (arrows): polymerase chain reaction (PCR), full blood counts, and immunological assays were performed. (B) Parasitemia was determined by quantitative PCR in participants infected with 1800 parasitized red blood cells (pRBC). The dotted line indicates the predetermined parasite treatment threshold of 1000 parasites/mL. Brackets represent the day of antimalarial treatment (Rx) on day 7 (*n* = 15) or day 8 (*n* = 24). The mean parasitemia ± the standard error is presented. (C) Longitudinal CD16^+^ DC spontaneous cytokine production and in response to pRBC stimulation during IBSM. Boolean gating of CD16^+^ DCs reveals 7 cytokine-producing populations. Shown are the relative proportion of each combination of TNF-, IL-12-, or IL-10-producing cells with (D) spontaneous cytokine production (No Stim) or in response to pRBC stimulation. Light gray circles represent day 0 (before infection) and dark gray circles represent day 7 (peak parasitemia). The Wilcoxon matched-pairs sign rank test was used to compare longitudinal data. Tests were 2-tailed and considered significant if *P* < .05. CD16^+^ DC cytokine production was evaluated according to the gating example in Figure 1.

To examine the specific impact of *P falciparum* on CD16^+^ DCs in vivo, whole blood was collected and processed from volunteers participating in an induced blood-stage *P. falciparum* malaria (IBSM) clinical trial. Volunteers were inoculated intravenously with ~1800 pRBC, and peripheral parasitemia was monitored by real-time polymerase chain reaction, until a predetermined threshold of ~1000 pRBC/mL was reached, at which point patients were treated with an antimalarial regimen [25] (Figure 4A and B). The median peak parasitemia was 4000 parasites per mL of blood (IQR, 700–10000). Before infection, 7% (IQR, 3%–12%) of CD16^+^ DCs spontaneously produced IL-10, IL-12, or TNF ex vivo, with TNF (5% [IQR, 4%–10%]) dominating the response. At peak parasitemia, spontaneous TNF cytokine production increased significantly to 15% (IQR, 10%–18%; *P* = .03), indicating that CD16^+^ DCs were activated in vivo during a first *P falciparum* infection (Figure 4C). This increased spontaneous cytokine production was driven by a significant increase in TNF single (*P* = .01) and TNF/IL-10 coproducing CD16^+^ DCs (*P* = .04) (Figure 4D). Next, we assessed the impact of *P falciparum* infection on CD16^+^ DCs ability to respond to pRBC in vitro restimulation. At peak parasitemia, there was a significant increase in TNF/IL-10 coproduction in response to in vitro pRBC restimulation (*P* = .04), with no significant changes in single TNF or IL-10 production (Figure 4C). There was no significant change in total CD16^+^ DC cytokine production in response to uRBC (Supplementary Figure 2). In response to TLR ligation, CD16^+^ DCs significantly increased IL-12 (TLR1/2, TLR4; *P* = .03) and IL-10 (TLR4; *P* = .03) but not TNF production (Supplementary Figure 2). Taken together, these data show that during subpatent induced blood-stage malaria, CD16^+^ DCs enhance spontaneous cytokine production as well as responsiveness to pRBC and TLR stimulation in vitro.

### CD16^+^ Dendritic Cells Are Activated in Induced Subpatent Blood-Stage Malaria

**Figure 5.**
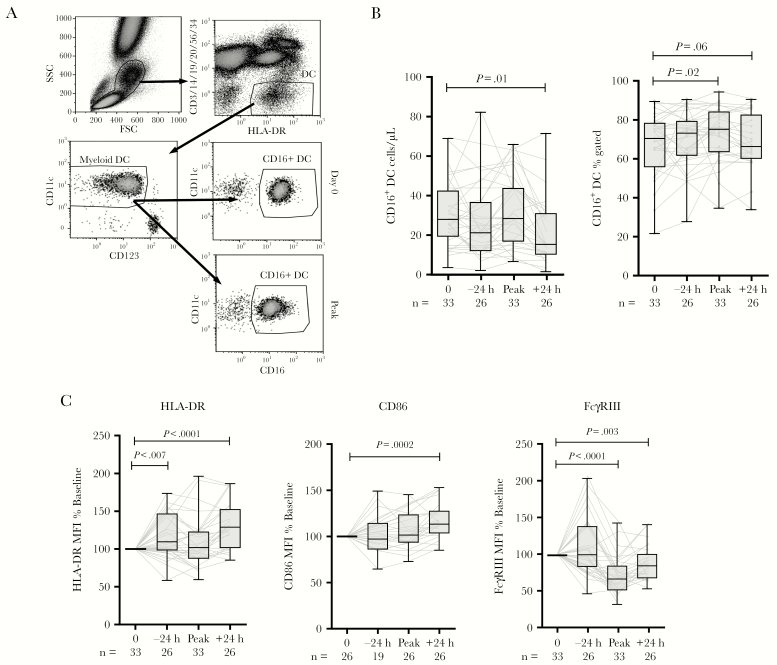
Induced blood-stage *Plasmodium falciparum* infection uniquely activates circulating CD16^+^ dendritic cells (DCs). CD16^+^ DCs were assessed longitudinally in volunteers. (A) Fresh whole blood gating strategy for CD16^+^ DCs. Myeloid DC subsets were identified as negative for lineage markers (CD3, CD14, CD19, CD20, CD34, and CD56), HLA-DR^+^ (2nd panel), CD11c^+^CD123^−^ (3rd panel), and CD16^+^ (4th and 5th panels). (B) The absolute number (1st panel) and proportion (2nd panel) of circulating CD16^+^ DCs during induced blood-stage *P falciparum* malaria (IBSM). (C) HLA-DR (1st panel), CD86 (2nd panel), and FcγRIII (3rd panel) expression on circulating CD16^+^ DCs during IBSM. Box plots show the 10th–90th percentile, median, and interquartile range for data from all participants. Data points outside of box plots represent patients that were outliers. The Wilcoxon matched-pairs signed-rank test was used to compare longitudinal data. Tests were 2-tailed and considered significant if *P* < .05. FSC, forward scatter; SSC, side scatter.

In addition to cytokine production, we examined the number, proportion, and activation of circulating CD16^+^ DCs in fresh whole blood (Figure 5A), from healthy malaria naive volunteers before, during induced blood stage malaria, and 24 hours after antimalarial drug treatment. At peak parasitemia, there was no change to the absolute number of CD16^+^ DCs in the blood (*P* = .8) (Figure 5B). However, the proportion of CD16^+^ DCs amongst the total myeloid DC compartment increased (*P* = .02) (Figure 5B), reflecting the loss of CD1c^+^ DCs in the blood [3]. CD16^+^ DC maturation and activation were evaluated by measuring changes in surface expression of antigen-presenting molecule major histocompatibility complex (MHC) class II (HLA-DR), costimulatory molecule CD86 (B7-2), and antibody receptor CD16 (FcγRIII). The loss of the latter has been associated with CD16^+^ DC activation [28]. During induced blood-stage malaria, we observed maturation and activation of CD16^+^ DCs with increased HLA-DR (*P* < .0001) and CD86 (*P* = .0002) expression and reduced CD16/FcγRIII expression (*P* = .003) (Figure 5C). The observed reduction in CD16/FcγRIII expression did not impede identification of CD16^+^ DCs in fresh whole blood (Figure 5A).

### Lack of CD16^+^ Dendritic Cell Activation in Previously Exposed Individuals Experiencing an Acute Malaria Episode

**Figure 6.**
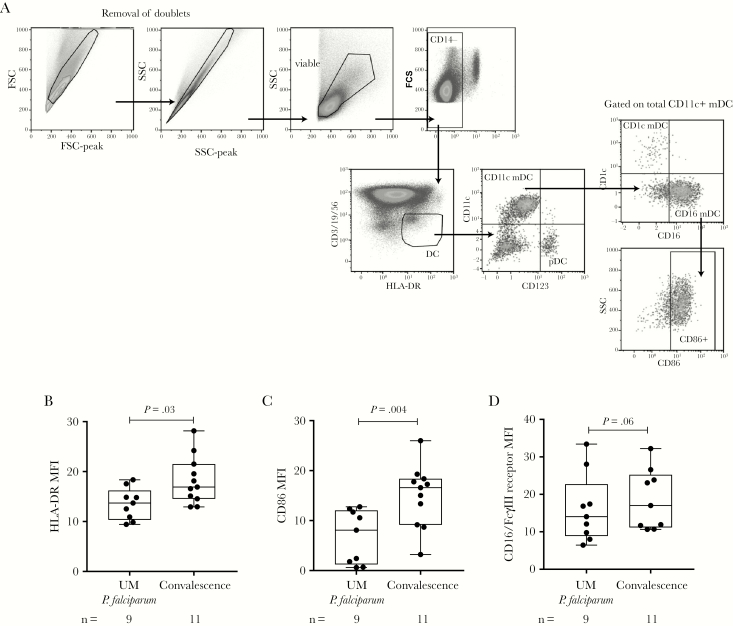
Peripheral blood mononuclear cell (PBMC) CD16^+^ dendritic cells (DCs) in adults with clinical *Plasmodium falciparum* malaria. Patients with uncomplicated *P falciparum* malaria (UM) were compared with age-matched convalescent adults from the same endemic region who were polymerase chain reaction negative for malaria parasites. (A) HLA-DR, (B) CD86, and (C) CD16 (FcγRIII) expression on CD16^+^ DCs in adults with acute uncomplicated malaria (UM) and at convalescence 28 days after antimalarial treatment. Box plots show the 10th-90th percentile, median, and interquartile range for data from all participants. Mann-Whitney *t* test was used for comparison between patients with acute infection and patients at convalescence (28 days after antimalarial treatment). MFI, median fluorescence intensity.

We then assessed CD16^+^ DCs in adults and children living in an area of perennial unstable malaria transmission, using cryopreserved PBMCs collected during an acute clinical episode of *P falciparum* malaria or during convalescence (28 days after malaria treatment and successful parasite clearance) (Table 1). The HLA-DR and CD86 expression were significantly diminished on CD16^+^ DCs in adults during acute malaria compared with those at convalescence (*P* = .03 and *P* = .004, respectively) (Figure 6B and C). In children, there was no significant difference between HLA-DR or CD86 expression between acute malaria and convalescent controls (Supplementary Figures 3A and 3B). CD16/FcγRIII expression on CD16^+^ DCs did not differ significantly between acute infection and convalescence in both children and adults (Figure 6C and Supplementary Figure 3C). At convalescence, children expressed less CD16/FcγRIII on CD16^+^ DCs than adults (*P* = .03) (Supplementary Figure 3C). Taken together, our findings show that during an acute malaria episode caused by natural *P falciparum* infection, CD16^+^ DC maturation appears impaired with reduced HLA-DR and CD86 but stable CD16 expression.

## DISCUSSION

Our results illustrate that CD16^+^ DCs undergo unique and distinctive activation in response to a primary subpatent *P falciparum* infection, with enhanced expression of HLA-DR and CD86, reduction in CD16/FcγRIII, and increased coproduction of IL-10 and TNF. CD16^+^ DC activation during induced *P falciparum* blood-stage malaria is consistent with the activation of this subset during induced *Plasmodium vivax* blood-stage malaria [30] and induced *P falciparum* sporozoite infection [23], suggesting pan *Plasmodium* activation of CD16^+^ DCs after *Plasmodium* infection in naive healthy adults. We have recently shown that (1) both CD1c^+^ DCs and plasmacytoid DCs are not activated during induced *P falciparum* or *P vivax* blood-stage malaria [3, 24] and (2) the rare classic CD141^+^ DC subset is drastically reduced in induced *P vivax* blood-stage malaria [30]. Together, our data define CD16^+^ DCs as the only blood DC subset activated during induced human *Plasmodium* malaria, suggesting that CD16^+^ DCs may be involved in the initiation of immune responses required for parasite control during a primary infection.

Using an indirect gating method, we characterized CD16^+^ DC cytokine production during subpatent *P falciparum* infection. We show that CD16^+^ DCs respond to TLR1/2 and TLR4 stimulation with robust TNF production both before and during induced blood-stage malaria. Tumor necrosis factor production in response to TLR stimulation is consistent with prior reports identifying CD16^+^ DCs as an inflammatory DC subset [20, 21], including a subpopulation of CD16^+^ DCs expressing the carbohydrate modification 6-sulfo LacNac (slan) [31]. Unlike monocytes, slanDCs show DC-like morphology in lymphoid tissue [32] and the ability activate T cells [32, 33], as reported for CD16^+^ DC [7]. Tumor necrosis factor is a major effector cytokine in malaria, with roles in both protection and pathogenesis. Studies in malaria patients identified monocytes [34], particularly CD14^+^CD16^+^ inflammatory monocytes [35], and γδ T cells [34], as the main cellular sources of TNF. Our data show that CD16^+^ DCs also contribute to TNF production in early infection and potentially during clinical malaria.

Interleukin-10 production by CD16^+^ DCs stimulated with *P falciparum* was proportionally greater compared with IL-10 responses induced by TLR stimulation, whereas the TNF response was comparatively modest. A recent report using isolated blood CD1c^+^ and CD141^+^ DCs showed no production of TNF, IL-10, or IL-12 by these DCs upon pRBC stimulation when compared with TLR4 stimulation [11]; however, CD16^+^ DCs were not included. From the data presented here, it is evident that CD16^+^ DCs do have the ability to respond to TLR and pRBC stimulation, yet with distinct cytokine profiles; setting them apart from classic blood DCs. In addition to increased spontaneous TNF production after *P falciparum* infection, CD16^+^ DCs became multipotent cytokine-producing cells increasing coproduction of TNF and IL-10, particularly upon *Plasmodium* re-exposure in vitro, which further skewed the CD16^+^ DC cytokine profile towards IL-10 production. Interleukin-10 producing DCs can contribute to immunoregulatory processes through the induction of highly suppressive regulatory T cells [36, 37] and IFN-γ^low^ IL-10^high^ type 1 regulatory (Tr1) cells [38]. We recently identified Tr1 cells in induced *P falciparum* infection, and parasite-specific IL-10 production by these cells suppresses proinflammatory cytokine production [39]. The parasite-induced IL-10 production by CD16^+^ DCs reported here suggests these DCs may have a role in the induction of regulatory cells in primary *P falciparum* infection. Such suppressive T-cell responses may blunt protective immune responses and result in higher parasite burden [40, 41], potentially further increasing CD16^+^ DC IL-10 production [42]. Together, our data suggest that during induced malaria, CD16^+^ DCs contribute to both inflammatory and regulatory immune processes. Further evaluation of cytokine production by CD16^+^ DCs and how they impact downstream immune responses is needed.

Data relating to CD16^+^ DC activation in human malaria are sparse. In this study, we show that the absolute number of circulating CD16^+^ DCs are stable, and, moreover, their proportion in the total myeloid DC compartment increased during induced blood-stage malaria. This relative increase is likely a reflection of the loss of peripheral CD1c^+^ DCs [3]. The increased CD16^+^ DC maturation during early subpatent *Plasmodium* infection is consistent with our recent report of CD16^+^ DCs being the only blood DC subset with increased HLA-DR expression during induced blood-stage *P vivax* malaria [30], suggesting that DC subsets are affected similarly by different *Plasmodium* species. High expression levels of HLA-DR are associated with efficient phagocytosis of *P falciparum*-infected RBCs [11] and essential for antigen presentation to T cells. The reduction in CD16/FcγRIII, which is internalized upon cross-linking by immune complexed antigen [28], together with the increased HLA-DR and CD86 expression on CD16^+^ DCs likely enhances their potential to activate CD4^+^ T cells. Increased CD16^+^ DC maturation has been reported in *P falciparum*-sporozoite volunteer infection studies [23], which mimics the natural route of *Plasmodium* infection by mosquito bite [43]. Similarities between results from the induced *P falciparum*-sporozoite infection [23], and data presented here, indicate that CD16^+^ DC maturation occurs in response to the early blood-stage of infection, independent of *Plasmodium* liver stages.

In contrast to primary subpatent infection, HLA-DR and CD86 expression were reduced on CD16^+^ DCs in adults with acute malaria when compared with convalescent controls. These findings are consistent with reports of reduced HLA-DR expression during acute malaria on CD1c^+^ DCs and CD141^+^ DCs in adults [44] and children [14, 45]. In children with acute malaria, we observed no significant difference in HLA-DR or CD86 expression when compared with convalescent samples. The less dramatic changes in CD16^+^ DC maturation in children compared with adults seen here suggest that CD16^+^ DC dysfunction may result from cumulative exposure. Differences in CD16^+^ DC maturation between primary induced infection in naive adults and naturally acquired acute malaria are not surprising, and many factors may influence these differences including the magnitude of parasite burden or prior malaria. Dendritic cell maturation can be suppressed in the presence of IL-10 [46, 47]. We have previously reported significantly increased plasma IL-10 in patients from the same endemic area [4, 48], whereas plasma IL-10 is not detectable in primary induced *P falciparum* infection [49]. These data together with the parasite-mediated IL-10 production by CD16^+^ DC suggest that increasing autocrine IL-10 production [42] or increased systemic IL-10 may impair DC maturation in acute malaria.

## CONCLUSIONS

Taken together, we describe for the first time the effect of *Plasmodium* parasites on human blood CD16^+^ DC function. We demonstrate that CD16^+^ DCs are uniquely activated by *P falciparum* stimulation and significantly increase spontaneous coproduction of TNF and IL-10 during a primary subpatent *P falciparum* infection. Our data, presented here and previously [3, 24], together reveal that CD16^+^ DCs are the only DC subset activated during induced blood-stage malaria. As dual cytokine producers, CD16^+^ DCs contribute to inflammatory as well as regulatory innate immune processes and are key responders during early subpatent *P falciparum* infection.

## Supplementary Data

Supplementary materials are available at *The Journal of Infectious Diseases* online. Consisting of data provided by the authors to benefit the reader, the posted materials are not copyedited and are the sole responsibility of the authors, so questions or comments should be addressed to the corresponding author.

## Supplementary Material

jiy555_suppl_Supplementary_Figures

## Notes

***Acknowledgments.*** We thank the Q-pharm staff who conducted the human blood-stage infection studies and in particular Dr. Suzanne Elliot, Nannette Douglas, and Gem Mackenroth for supporting the research. We thank Dr. Paul Griffin for advice and technical assistance. We thank all staff with the Papuan Community Health and Development Foundation and all teams involved in the Timika clinical malaria studies. We thank the Australian Red Cross Blood Service for their support. We thank the volunteers who participated in all clinical trials.

***Author contributions.*** J. R. L., G. M., and T. W. conceived and designed the experiments and prepared the manuscript. J. R. L., G. M., T. W., and P. E. T. performed the experiments with assistance from K. A. P. and F. H. A. J. R. L., G. M., T. W., and M. J. B. analyzed and interpreted the data. E. K., N. M. A., and R. N. P. conducted the clinical malaria studies. N. M. A., C. R. E., and M. J. B. provided intellectual input and assisted with manuscript preparation. J. S. M. conducted the induced blood-stage *Plasmodium falciparum* malaria (IBSM) clinical trial and assisted in manuscript preparation.

***Disclaimer.*** The views expressed in this publication are those of the authors and do not reflect the views of the National Health and Medical Research Council (NHMRC). The funders had no role in study design, data collection and analysis, decision to publish or preparation of the manuscript.

***Finanical support.*** This work was funded by the National Health and Medical Research Council (NHMRC) (Project Grant 1021198, Program Grant 1037304, and Fellowships to M. J. B. [GNT1071736 and GNT1141632], N. M. A. [1135820], J. S. M., C. R. E., and G. M.), Channel 7 Children’s Research Foundation (Grant 151016), and an APA PhD Scholarship to J. R. L. The clinical trials that enabled sample collection were funded by Medicines for Malaria Venture. R. N. P. is a Wellcome Trust Senior Fellow in Clinical Science (200909). The Timika Malaria Research Unit is supported by the Australian Department of Foreign Affairs.

***Potential conflicts of interest.*** All authors: No reported conflicts of interest. All authors have submitted the ICMJE Form for Disclosure of Potential Conflicts of Interest.
