# Supplementary material for: Plasmodium falciparum Activates CD16+ Dendritic Cells to Produce Tumor Necrosis Factor and Interleukin-10 in Subpatent Malaria
Source: J Infect Dis. 2018 Oct 15;219(4):660–71. doi: 10.1093/infdis/jiy555 (PMC6339523; doi:10.1093/infdis/jiy555)
Supplement: jiy555_suppl_Supplementary_Figures [file jiy555_suppl_supplementary_figures.pdf]

Plasmodium falciparum activates CD16^{+} dendritic cells to produce TNF and IL-10 in human volunteers

Jessica R. Loughland[1*], Tonia Woodberry[1¥], Michelle J Boyle[1,2], Peta E. Tipping[1€], Kim A. Piera[1], Fiona H. Amante[3], Enny Kenangalem[4,5], Ric N. Price[1], Christian R. Engwerda[3], Nicholas M. Anstey[1], James S. McCarthy[3¶], Gabriela Minigo[1*¶].

[1]Menzies School of Health Research, Darwin, Australia and Charles Darwin University, Darwin, Australia; [2] Burnet Institute, Melbourne, VIC, Australia; [3]QIMR Berghofer Medical Research Institute, Brisbane, Australia; [4]Timika Malaria Research Program, Papuan Health and Community Development Foundation, Timika, Papua, Indonesia; [5]District Health Authority, Timika, Papua, Indonesia.

€ Present Address: Royal Darwin Hospital, Darwin, Australia.

¥ Present Address: The Australian National University, Australia.

¶ These authors contributed equally to this work.

* Corresponding Authors

Running Title:

CD16^{+} DC activate in malaria

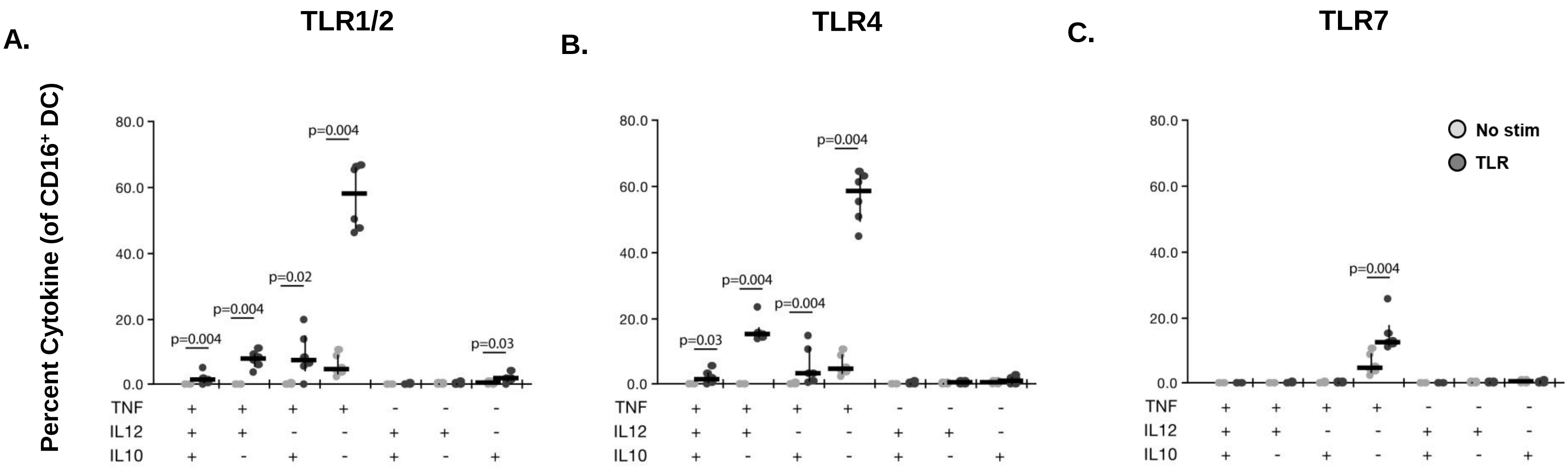

Supplementary Figure 1 Boolean gating of CD16⁺ DC cytokine response to in-vitro TLR stimulation. Boolean gating of CD16⁺ DCs revealed seven cytokine-producing populations. Shown are the relative proportion of each combination of TNF, IL-12 or IL-10-producing cells after **A.** TLR1/2, **B.** TLR4 or **C.** TLR7 stimulation. TLR stimulations (dark grey circles) were compared to non-stimulation controls (light grey circles). The Mann-Whitney t-test was used for comparison between ‘no-stim’ and TLR. Tests were two-tailed and considered significant if *p*-values <0.05.

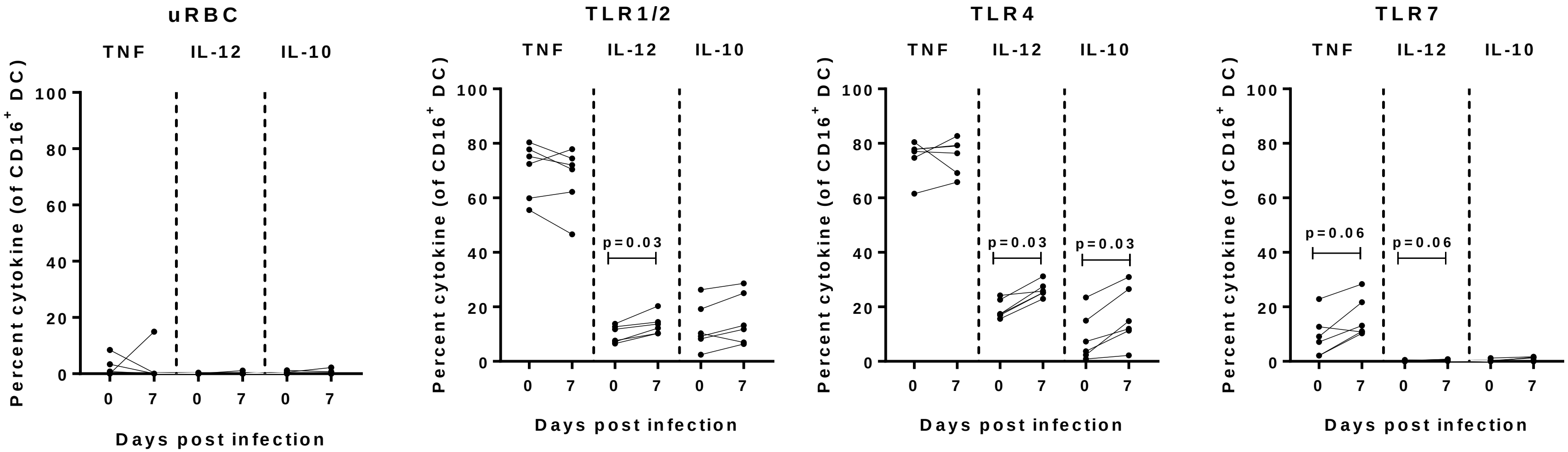

Supplementary Figure 2 CD16+ DC cytokine production in response to uRBC or TLR stimulation, before and during IBSM. Longitudinal CD16+ DC TNF, IL-12 or IL-10 cytokine production in response to uRBC, TLR1/2, TLR4 and TLR7 stimulation, before infection (day 0) and at peak parasitemia (day 7). The Wilcoxon matched-pairs sign rank test was used to compare longitudinal data. Tests were two-tailed and considered significant if *p*-values <0.05.

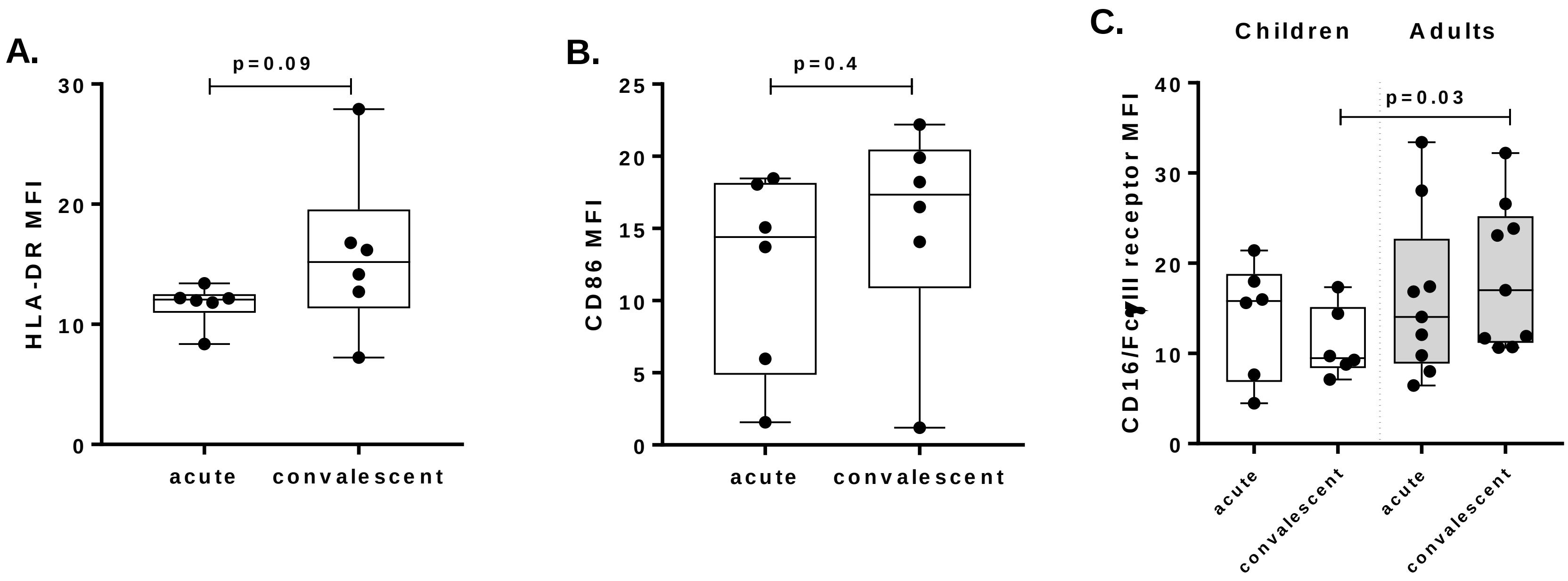

Supplementary Figure 3 PBMC CD16$^+$ DCs in children with clinical *P. falciparum* malaria. **A.** HLA-DR, **B.** CD86 and **C.** CD16 (FcγRIII) expression on CD16$^+$ DCs in children with acute uncomplicated malaria (UM) and at convalescence 28 days post anti-malarial treatment. Box plots show the 10th-90th percentile, median and interquartile range for data from all participants. The Mann-Whitney t-test was used for comparison between patients with acute infection and patients at convalescence (28 days post anti-malarial treatment). Abbreviations: MFI, median fluorescence intensity.
